# Supplementary material for: End Sequence Analysis Toolkit (ESAT) expands the extractable information from single-cell RNA-seq data
Source: Genome Res. 2016 Oct;26(10):1397–410. doi: 10.1101/gr.207902.116 (PMC5052061; doi:10.1101/gr.207902.116)
Supplement: Supplemental Material [file supp_gr.207902.116_Supp_Materials.tar.gz › ESAT-esat_v0.1/doc/html/namespaceumms_1_1core_1_1model_1_1score.html]

ESAT: Package umms.core.model.score


ESAT


- Main Page
- Related Pages
- Packages
- Classes
- Files

- Packages

All Classes Namespaces Files Functions Variables Enumerator Pages

- umms
- core
- model
- score

Classes

Package umms.core.model.score

|  |  |
| --- | --- |
| Classes | |
| class | AbstractListScore |
|  | |
| class | BasicScanStatisticScore |
|  | |
| class | BinomialEnrichmentScore |
|  | |
| class | CountScore |
|  | |
| class | CoverageScore |
|  | |
| class | EndInWindowCountScore |
|  | |
| class | LengthScore |
|  | |
| class | MultiScore |
|  | |
| class | NewRatioScore |
|  | |
| class | PoissonEnrichmentScore |
|  | |
| class | RatioScore |
|  | |
| class | ScanStatisticScore |
|  | |
| class | StartInWindowCountScore |
|  | |
| class | SumScore |
|  | |
| class | WindowAllScore |
|  | |
| interface | WindowProcessor< S extends WindowScore > |
|  | |
| interface | WindowScore |
|  | |
| class | WindowScoreIterator< T extends WindowScore > |
|  | |


---

Generated on Fri Aug 15 2014 15:11:11 for ESAT by  

 1.8.7
